# Effect of the stacking order, annealing temperature and atmosphere on crystal phase and optical properties of Cu_2_SnS_3_

**DOI:** 10.1038/s41598-022-12045-3

**Published:** 2022-05-13

**Authors:** M. Y. Zaki, F. Sava, I. D. Simandan, A. T. Buruiana, C. Mihai, A. Velea, A. C. Galca

**Affiliations:** 1 National Institute of Materials Physics, Atomistilor 405A, 077125 Magurele, Romania; 2 Faculty of Physics, University of Bucharest, Atomistilor 405, 077125 Magurele, Romania

**Keywords:** Applied physics, Condensed-matter physics, Optical physics, Condensed-matter physics, Materials for devices, Materials for energy and catalysis

## Abstract

Cu_2_SnS_3_ (CTS) is emerging as a promising absorber for the next generation thin film solar cells (TFSC) due to its excellent optical and electronic properties, earth-abundance and eco-friendly elemental composition. In addition, CTS can be used as precursor films for the Cu_2_ZnSnS_4_ (CZTS) synthesis. The optical properties of CTS are influenced by stoichiometry, crystalline structure, secondary phases and crystallite size. Routes for obtaining CTS films with optimized properties for TFSC are still being sought. Here, the CTS thin films synthesized by magnetron sputtering on soda lime glass (SLG) using Cu and SnS_2_ targets in two different stacks, were studied. The SLG\Cu\SnS_2_ and SLG\SnS_2_\Cu stacks were annealed in S and Sn + S atmospheres, at various temperatures. Both stacks show a polymorphic structure, and higher annealing temperatures favor the monoclinic CTS phase formation. Morphology is influenced by the stacking order since a SnS_2_ top layer generates several voids on the surface due to the evaporation of SnS, while a Cu top layer provides uniform and void-free surfaces. The films in the copper-capped stack annealed under Sn + S atmosphere have the best structural, morphological, compositional and optical properties, with tunable band gaps between 1.18 and 1.37 eV. Remarkably, secondary phases are present only in a very low percent (< 3.5%) in samples annealed at higher temperatures. This new synthesis strategy opens the way for obtaining CTS thin films for solar cell applications, that can be used also as intermediary stage for CZTS synthesis.

## Introduction

Recently the development of new absorber materials for the substitution of the currently commercialized Cu(In,Ga)Se_2_ (CIGS) and cadmium telluride (CdTe) based solar cells has gained increasing interest^1^. These alloys are composed of expensive and scarce (In, Ga, Te), or toxic (Cd, Se) elements^2^. To overcome these issues, new earth-abundant and non-toxic materials are being subject to extensive research. Among these materials, the kesterite family Cu_2_ZnSn(S,Se)_4_ (CZT(S,Se)) has shown great potential due to their excellent optical properties, such as a direct optical gap between 1 and 1.5 eV and an absorption coefficient greater than 10^4^ cm^–1^^3,4^. However, their relatively modest power conversion efficiency (PCE), of 12.7% in the case of CZTSSe for example^5^, is still very low compared to the above 20% for CIGS-based solar cells^6^. Another important disadvantage is the difficulty to obtain films with no secondary phases. Several binary and ternary sulfides, such as ZnS, Sn_x_S_y_ and Cu_x_SnS_y_ are formed during the synthesis of these semiconductors^7,8^.

The ternary copper tin sulfide family (Cu_x_SnS_y_) includes Cu_2_SnS_3_, Cu_3_SnS_4_, Cu_4_SnS_4_, Cu_2_Sn_3_S_7_ and Cu_7_Sn_3_S_8_^9,10^ crystalline compounds, and some of them are also regarded as a potential absorber layers for thin film solar cells (TFSC). Among these compounds, Cu_2_SnS_3_ (CTS) has attracted particular attention due to its potential to be used as small band semiconductors for infrared photodetectors, but also for photocatalytic, optoelectronic, and supercapacitor applications^11–16^. CTS is also regarded as a precursor for the synthesis of CZTS materials used in solar cells devices^17,18^. A highly crystalline CZTS phase can be obtained by the optimization of the Cu_2_SnS_3_ synthesis conditions and the addition of a ZnS layer^19^ on top of CTS. Further sulfurization of the CTS\ZnS stacked layers at high temperatures will produce a reaction between the two films and the formation a single CZTS^20^ phase. This synthesis route is regarded as an optimal solution to avoid the formation of CTS and ZnS as secondary phases during the growth of CZTS^21^. On the other hand, CTS is a p-type semiconductor with a high absorption coefficient and a suitable band gap (0.9–1.40 eV) that can be used as an absorber layer in TFSC^22–24^. The tuning of the band gap is possible due to the polymorphic nature of the CTS material^25^, which crystallizes in three structures: cubic (E_g_ = 0.96 eV), monoclinic (E_g_ = 0.94 eV), and tetragonal (E_g_ = 1.35 eV)^26–28^. Usually, CTS crystallizes in the tetragonal structure at temperatures around 350 °C^29^. On the other hand, a sulfurization process above 400 °C, needed to enlarge the crystallites, leads to the formation of cubic and/or monoclinic CTS^30^. Finally, the used precursors, the synthesis technique and process can also influence the final structure of the CTS films^26^. Various physical and chemical techniques have been employed to synthesize CTS films, such as pulsed laser deposition (PLD)^30^, spray pyrolysis^31^, thermal evaporation^32^, chemical bath deposition (CBD)^19^, solvothermal synthesis^33^, wet chemical method^29^ and magnetron sputtering^34^. Magnetron sputtering is a physical technique for the synthesis of large-area and highly uniform films with controllable thickness and composition. Parameters such as pressure inside the deposition chamber, power applied to the magnetrons, deposition rate and time can be varied to optimize the synthesis conditions to obtain high-quality films.

Recently, works on CTS materials have raised and several groups of researchers are trying to synthesize CTS films with the desired properties by exploring different deposition techniques and growth parameters. Suryawanshi et al.^35^ studied the effect of the thickness (by varying the deposition time) on the properties of CBD deposited CTS thin films. The structural studies revealed the formation of tetragonal CTS along with the presence of Cu_2_S secondary phase. The increase in film thickness showed an improvement in crystallinity, while the composition of the films was Cu-rich. The obtained band gap values were higher than expected, ranging from 1.8 to 2.7 eV. The influence of the quantity of sulfur on CTS materials was investigated by Sozak et al.^36^ It was found that the films with a high amount of sulfur contain less secondary phases and enhanced surface morphology, leading to tetragonal CTS films with optimal band gap. Liu et al.^37^ investigated the effect of sulfurization time on CTS based solar cells. A longer annealing time helps obtaining more uniform, denser and larger crystallites. SnS_2_ secondary phase was detected in the films annealing for shorter times, while longer sulfurization leads to the formation of Cu_1.81_S crystalline phase resulting from the decomposition of CTS. Films annealed for 20 min showed the best structural, morphological and compositional properties leading to a solar cell device with a PCE of 3.68%. The milling time influences the mechanochemical obtained CTS nanocrystals as found by Trajic et al.^38^ Raman analysis revealed the formation of monoclinic and tetragonal CTS phases. However, CuS and SnS secondary phases were also observed. Short milling time helps suppressing Sn-based secondary phases, while for removing Cu-based phases high milling time is needed. An increase in CTS content is observed up to 15 min milling, while for longer milling time the CTS phase degrades.

The aim of this study is to explore new synthesis routes for polycrystalline Cu_2_SnS_3_ thin films with optimized physico-chemical properties for photovoltaic applications, more specifically, that can be used in the future research^39^, as precursors for the synthesis of CZTS films, by annealing CTS\ZnS stacks. The tuning of the crystallographic phase and stoichiometry of nanocrystalline CTS layers by means of thermal annealing at various temperatures and in different atmospheres is investigated. Finally, the optical properties of the obtained films are explored as a function of the annealing temperature and atmosphere, different sample stacking structures and stoichiometries.

## Results and discussion

**Figure 1:**
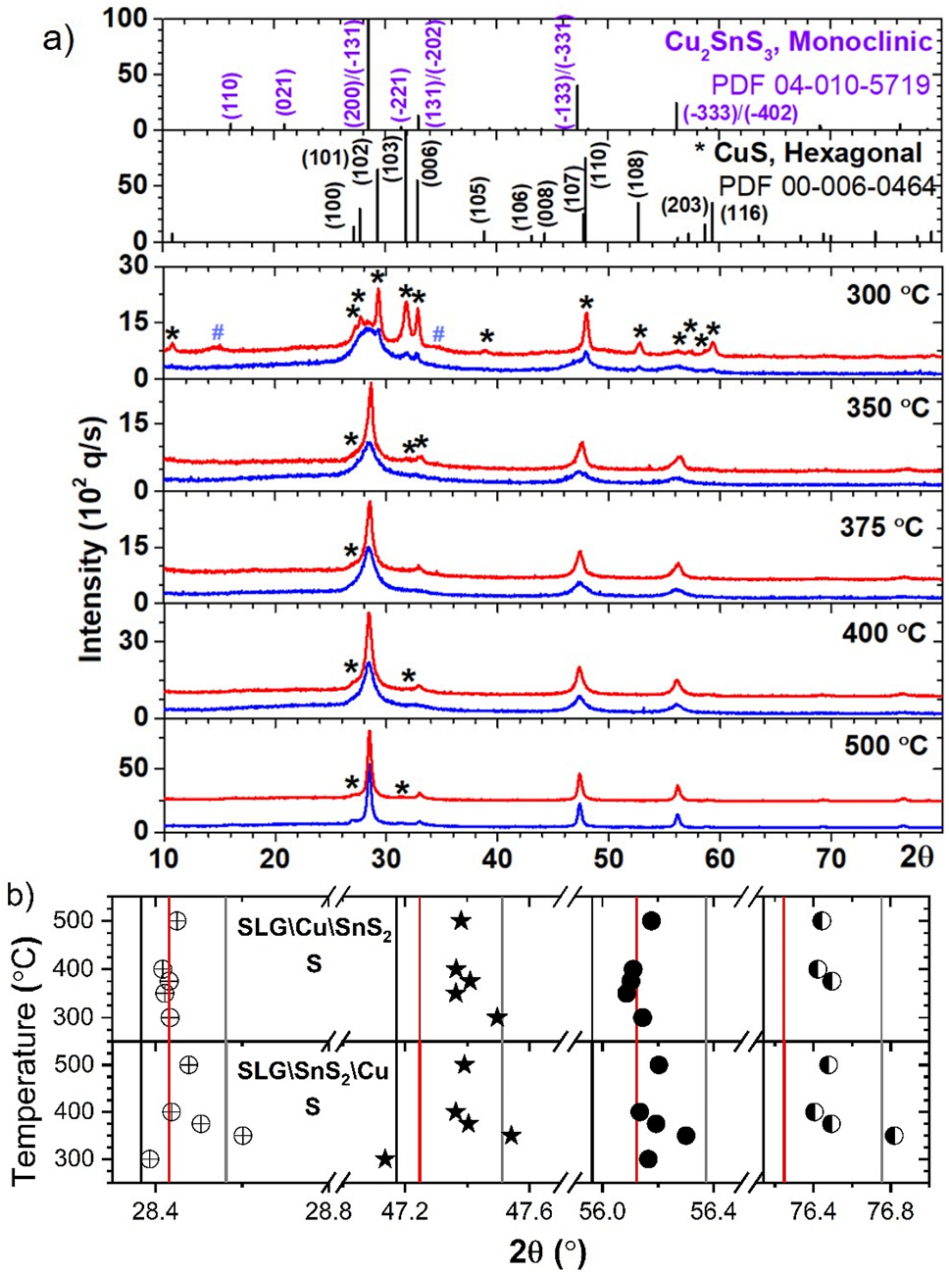
GIXRD analysis of the stacks annealed in sulfur atmosphere at 300, 350, 375, 400 and 500 °C. (**a**) GIXRD patterns of the SLG\Cu\SnS_2_ stack (blue) and the SLG\SnS_2_\Cu stack (red, shifted vertically for a better representation). The ICDD patterns of monoclinic CTS (04-010-5719) and hexagonal CuS (00-006-0464) are also shown. There are some traces of crystalline CuSn_3.75_S_8_ PDF 04-008-7270 (~ 3%) marked as “#” in the SLG\SnS_2_\Cu stack annealed in sulfur atmosphere at 300 °C. (**b**) The angular positions of the most intense CTS peaks in relation to the ICDD cards of tetragonal (04-009-7947) (gray: (112), (204)/(220), (116)/(312), (316)/(332)), cubic (04-002-6009) (black: (111), (220), (311), (331)) and monoclinic (04-010-5719) (red: (200)/(−131), (−133)/(−331), (−333)/(−402), (−135)/(−531)) structures.

Figure 1a shows the grazing incidence X-ray diffraction (GIXRD) results of the SLG\Cu\SnS_2_ and SLG\SnS_2_\Cu stacks annealed at different temperatures (300, 350, 375, 400 and 500 °C) in sulfur atmosphere. The as-deposited samples are fully amorphous (data not shown) which means that the stacked films (Cu and SnS_2_) are almost diffused^40^ after deposition. The sulfurization at 300 °C produces a slow formation of crystalline Cu_2_SnS_3_ nanoclusters (the diffraction peaks of the CTS phase are broad), as compared with the much faster reaction of sulfur atoms (from vapors or from SnS_2_) with Cu atoms, which produces much larger clusters (sharper diffraction peaks) of the polycrystalline hexagonal CuS (h-CuS) phase^41^, space group P63/mmc (194) (ICDD 00–006-0464). In the SLG\SnS_2_\Cu stack, h-CuS is the dominant phase, while in the SLG\Cu\SnS_2_ stack, h-CuS is a minor phase, which means that it is produced mainly by the sulfur atoms from atmosphere. In addition, few peaks of the CuSn_3.75_S_8_ crystalline phase are also observed. This behavior is maintained by sulfurization at 350 °C, but the formation of CTS crystalline clusters is faster (the diffraction peaks of the CTS phase are much sharper) than the formation of h-CuS, and therefore the ratio of h-CuS phase is smaller in the SLG\SnS_2_\Cu stack, while in the SLG\Cu\SnS_2_ stack, only traces of h-CuS are observed (identified by a small broad shoulder at 2θ = 27.1°). After annealing at 375 °C, h-CuS is barely visible in both stacks. At 400 °C, the same low quantity of h-CuS phase is maintained in the SLG\Cu\SnS_2_ stack, while for the SLG\SnS_2_\Cu stack, h-CuS phase increases. After the final sulfurization at 500 °C, h-CuS increases again in both stacks, probably due to low Sn content in the films as a consequence of SnS evaporation^42,43^. However, this low quantity secondary h-CuS phase is usually on the surface of the films and can be removed by KCN etching^44^.

To analyze the polymorphism of CTS, in Fig. 1b are synthetically presented the angular positions of the most intense CTS peaks in relation to the ICDD cards of cubic CTS (CTS_c_) (04-002-6009)^45^, monoclinic CTS (CTS_m_) (04-010-5719)^46^, and tetragonal CTS (CTS_t_) (04-009-7947)^47^ structures. For the SLG\Cu\SnS_2_ stack sulfurized at 300 °C, the positions of the most intense peaks are proportionally in relation with the monoclinic and tetragonal references. Between 350 and 500 °C, the main peaks are either a monoclinic structure or shifted between the CTS_t_ and CTS_m_ structures. For the SLG\SnS_2_\Cu stack, sulfurization at 300 °C produced a mixed CTS_c_-CTS_m_ phase, at 350 °C the CTS_t_ phase is formed, while between 375 and 500 °C the peaks are located between CTS_t_ and CTS_m_ structures. The monoclinic phase is considered to have lower defects and should be preferred as the highest solar cell PCEs are obtained in CTS absorber layers with this crystalline structure^48^.

**Figure 2:**
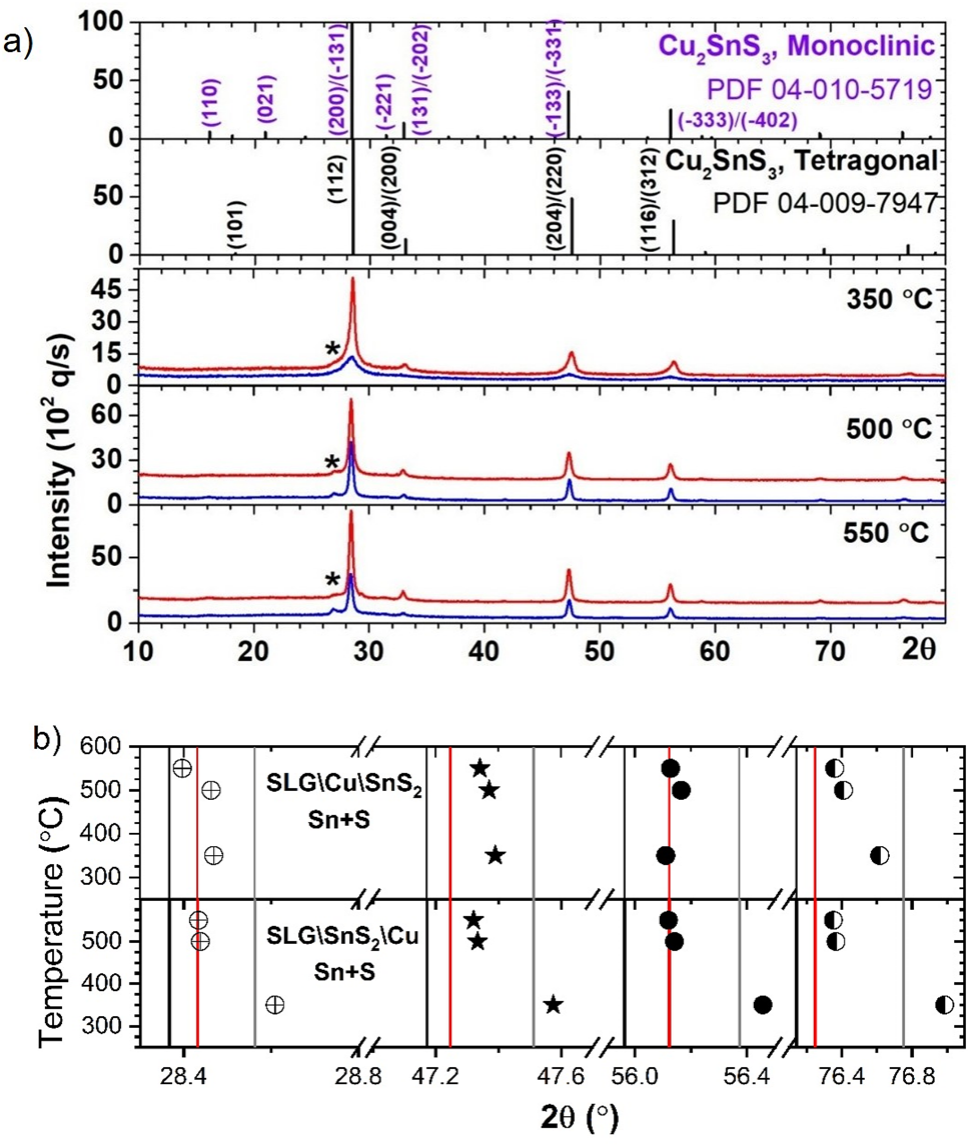
GIXRD analysis of the stacks annealed in sulfur and tin atmosphere at 350, 500 and 550 °C. (**a**) GIXRD patterns of the SLG\Cu\SnS_2_ stack (blue) and the SLG\SnS_2_\Cu stack (red, shifted vertically for a better representation). The ICDD patterns of monoclinic (04-010-5719) and tetragonal (04-009-7947) CTS are also shown. Traces of crystalline CuS (00-006-0464) phase marked as “*” are present in all the samples. (**b**) The angular positions of the most intense CTS peaks in relation to the ICDD cards of tetragonal (04-009-7947) (gray: (112), (204)/(220), (116)/(312), (316)/(332)), cubic (04-002-6009) (black: (111), (220), (311), (331)) and monoclinic (04-010-5719) (red: (200)/(−131), (−133)/(−331), (−333)/(−402), (−135)/(−531)) structures.

Annealing using Sn + S vapors, was performed on both stacks at three different temperatures: 350, 500 and 550 °C. Figure 2 shows the GIXRD results. The lowest annealing temperature (350 °C) produces different results in the two stacks. Monoclinic and tetragonal CTS peaks are observed in SLG\Cu\SnS_2_ Sn + S, while in SLG\SnS_2_\Cu Sn + S a single CTS_t_ structure is detected. At 500 °C and 550 °C, for both stacks, the CTS_m_ phase is formed. However, it is difficult to firmly discern between the three structures due to their neighboring XRD patterns^16,49^. Also, in all the samples, traces of the h-CuS phase (2θ = 27.1°) are identified, as a result of the evaporation of the volatile SnS and the decomposition of the CTS phase^34^. Thus, in the SLG\Cu\SnS_2_ stack the intensity of the CTS peak at 2θ = 28.5° is decreasing with increasing temperature, while the h-CuS peak at 2θ = 27.1° became more prominent. On the other hand, in the SLG\SnS_2_\Cu stack the presence of the h-CuS phase is less evident. Therefore, a second structural technique, such as Raman spectroscopy is needed.

**Figure 3:**
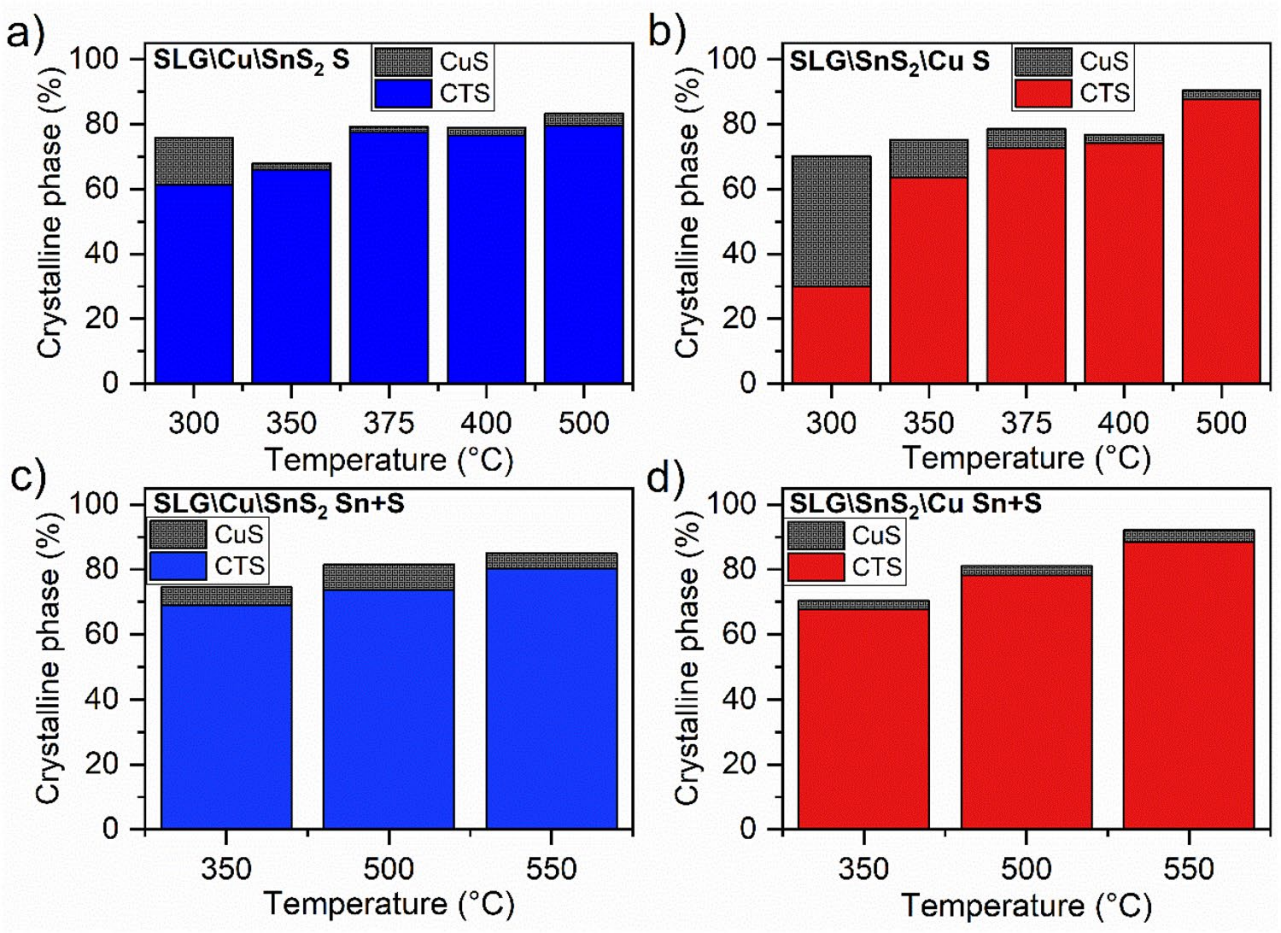
Crystalline CTS (tetragonal, cubic or monoclinic) and CuS phases percentages of the stacks annealed at different temperatures. (**a**) SLG\Cu\SnS_2_ S, (**b**) SLG\SnS_2_\Cu S, (**c**) SLG\Cu\SnS_2_ Sn + S, (**d**) SLG\SnS_2_\Cu Sn + S.

The crystalline phases percentages were determined from GIXRD patterns. Crystallinity can be estimated by subtracting the sum of the crystalline peaks areas from the total area of the diffraction pattern^50^. Figure 3 exhibits the results obtained using this method, for the two stacks annealed in S and Sn + S atmospheres. The CTS phase percentage is as low as 61.23% and 29.91% in the SLG\Cu\SnS_2_ S and SLG\SnS_2_\Cu S stacks, annealed at 300 °C. However, the CuS phase is more present in the sample with copper on top, which can be explained by the low annealing temperature, that causes the reaction of the Cu top layer with sulfur to form the CuS phase and the diffusion of a low amount of this phase to the SnS_2_ bottom layer. There is a significant amorphous phase in the samples as can be observed in Fig. 3 from the difference between 100% and the percentage of crystalline phases. The sulfurization at 350 °C leads to an enhancement of the CTS crystallinity in both sample sets. The presence of the CuS phase is very low (2.06%) in the SnS_2_-capped sample, while in the Cu-capped film, it is 11.5%. By increasing the temperature to 400 °C the CuS phase decomposes, leading to an enhancement of CTS crystallinity percentage in the two sample stacks^51^. At 500 °C, the Cu_2_SnS_3_ crystalline phase reaches the maximum value of 79.52% in the SLG\Cu\SnS_2_ S sample and 87.71% in the SLG\SnS_2_\Cu S film, while CuS also increases. The two other series of samples annealed using Sn + S atmosphere show similar results. At 350 °C, the CTS phase crystallinity percentages are 69.11% and 67.88% in SLG\Cu\SnS_2_ Sn + S and SLG\SnS_2_\Cu Sn + S, respectively, along with the presence of the CuS phase in very low percentages (5.47% and 2.53%, respectively). As the temperature increased to 500 and 550 °C, the percentage of the CTS crystalline phase improved^31^. The CuS phase percentages slightly increased and then decreased in the SLG\Cu\SnS_2_ Sn + S stack, while in the SLG\SnS_2_\Cu Sn + S stack only small differences are noticed (between 2.5 and 3.5%). The CTS crystallinity in the SLG\Cu\SnS_2_ Sn + S and SLG\SnS_2_\Cu Sn + S samples reached a maximum of 80.43% and 88.51%, respectively, when annealed at 550 °C under Sn + S atmosphere.

**Figure 4:**
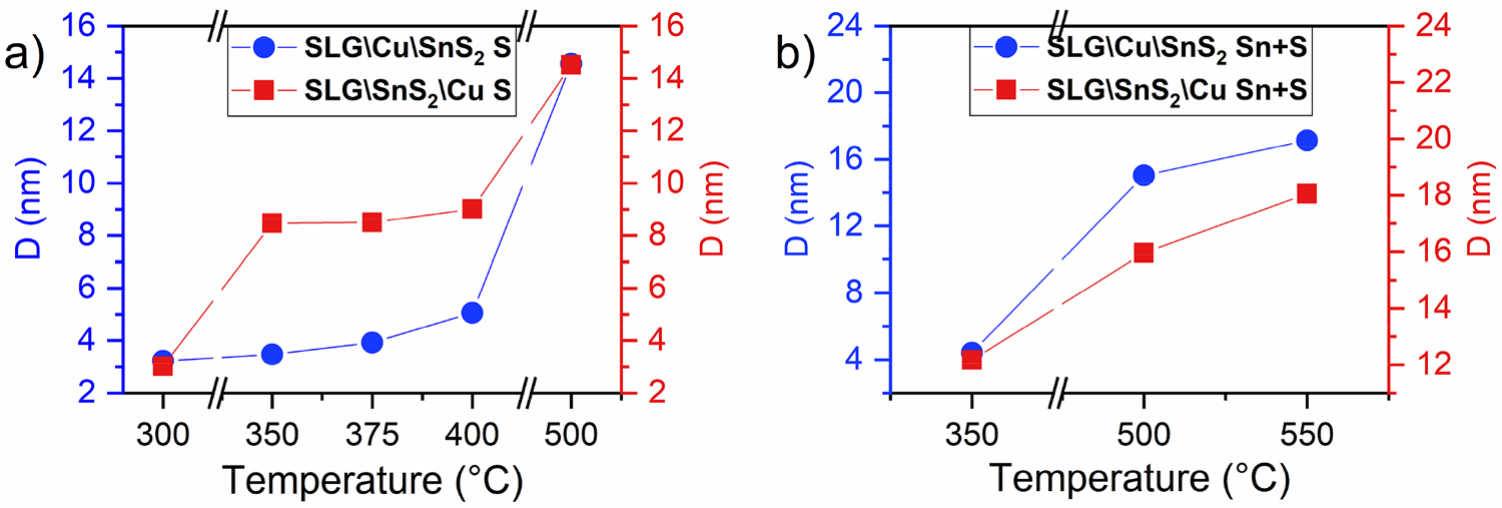
Average crystallite size in the SLG\Cu\SnS_2_ (blue) and SLG\SnS_2_\Cu (red) stacks. Samples were annealed under (**a**) S and (**b**) Sn + S atmospheres.

The average crystallite size of the CTS films prepared using two different stacks annealed under S and Sn + S atmospheres was calculated using the Scherrer equation^52^, and represented in Fig. 4. It was found that the crystallite size is increasing with the temperature increase^31^, regardless of the used stack or annealing atmosphere. The crystallite size of both stacks SLG\Cu\SnS_2_ S and SLG\SnS_2_\Cu S annealed using sulfur are between 3 and 15 nm for temperatures between 300 and 500 °C, respectively. Regarding the samples annealed under Sn + S atmosphere, the average crystallite size is increasing from 5 to 17 nm for the SLG\Cu\SnS_2_ Sn + S stack, and from 12 to 18 nm for the SLG\SnS_2_\Cu Sn + S stack, when the temperature increased from 350 to 550 °C.

As discussed, the Cu_2_SnS_3_ phase is known to be polymorphic and crystallize in monoclinic, cubic or tetragonal structures. It is very difficult to discern between the three CTS structures from GIXRD, especially between the CTS_m_ and CTS_c_, when the peaks are broad due to nanometric size of the crystallites, as in our samples. Therefore, Raman spectroscopy was employed as a complementary investigation tool. However, there are still controversies in the assignment of the Raman modes of the three Cu_2_SnS_3_ structures in the literature (see Table 1). Although several reports agree in the identification of the main Raman peak of each structure (i.e. 290 cm^−1^ for CTS_m_, 303 cm^−1^ for CTS_c_ and 337 cm^−1^ for CTS_t_)^26,53,54^, numerous contradictions have been reported in the assignment of other modes related to the three CTS structures. These confusions concern mainly the modes at 351 cm^−1^ that was attributed either to CTS_c_^27^ or to CTS_t_^55–57^, and at 352 cm^−1^ assigned to CTS_m_^19,26,29,49,57,58^ or to CTS_t_ structure^59^. Also, the peak located at 354 cm^−1^ was identified belonging to monoclinic structure of the CTS phase^50,54,60,61^, while Raadik et al. described this mode as a signature of CTS_c_^30^. Moreover, Oliva et al. assigned the peak at 356 cm^−1^ to CTS_t_ phase^26^, while Fernandes et al. stated that it belongs to the CTS_c_ structure^59^. On the other hand, the peak between 292 and 295 cm^−1^ was assigned to Cu_2_SnS_3_ by some authors^50,60^, while others attributed it to the Cu_3_SnS_4_ phase^62,63^. The same observation can be made for the peaks in the Raman shift 370–374 cm^−1^ identified as CTS_m_^29,30,49^, while other studies stated it is characteristic of the Cu_2_Sn_3_S_7_ phase^58,64^. Furthermore, the Raman mode in the region 314 − 318 cm^−1^ is even more controversial, since it can belong to Cu_2_SnS_3_^65^, Cu_2_Sn_3_S_7_^64^, Cu_4_SnS_4_^64^ or SnS_2_^66^.

**Table 1:** Raman peaks of the CTS_c_, CTS_m_ and CTS_t_ structures reported in the literature with the principal peaks being bolded.

CTS_c_ (cm^−1^)	CTS_m_ (cm^−1^)	CTS_t_ (cm^−1^)	References
267, 302, 354	222, 254, 292, 317, 350, 371	–	^30^
**303**, 365	255, 258, **290**–295, 349, 354	330, **337**	^50^
–	**290**–295, 354–357	–	^60^
**303**, 355	**290**, 295, 350, 354	**337**	^54^
–	**290**, 354	–	^61^
–	**290**, 346	286, 343	^17^
299, 351	**290**, 351	317, 338	^27^
–	**290**, 314, 352, 374	–	^58^
**303**, 355	291, 352	–	^29^
267, **303**, 355–356	224, 250, 268, **290**–293, 314–317, 352–353, 374	297, **337**, 356	^26^
–	**290**, 315, 352	289, 300, 335, 353	^19^
355	291–292	331, 335, 351	^55^
–	287, **290**, 348, 352, 354	330	^53^
**303**, 355	–	336–**337**, 351	^56^
267, **303**, 356	–	297, **337**, 352	^59^
**303**, 355	287, **290**, 352	297, 334, 351	^57^
–	225, **290**, 352, 371	335	^49^

**Figure 5:**
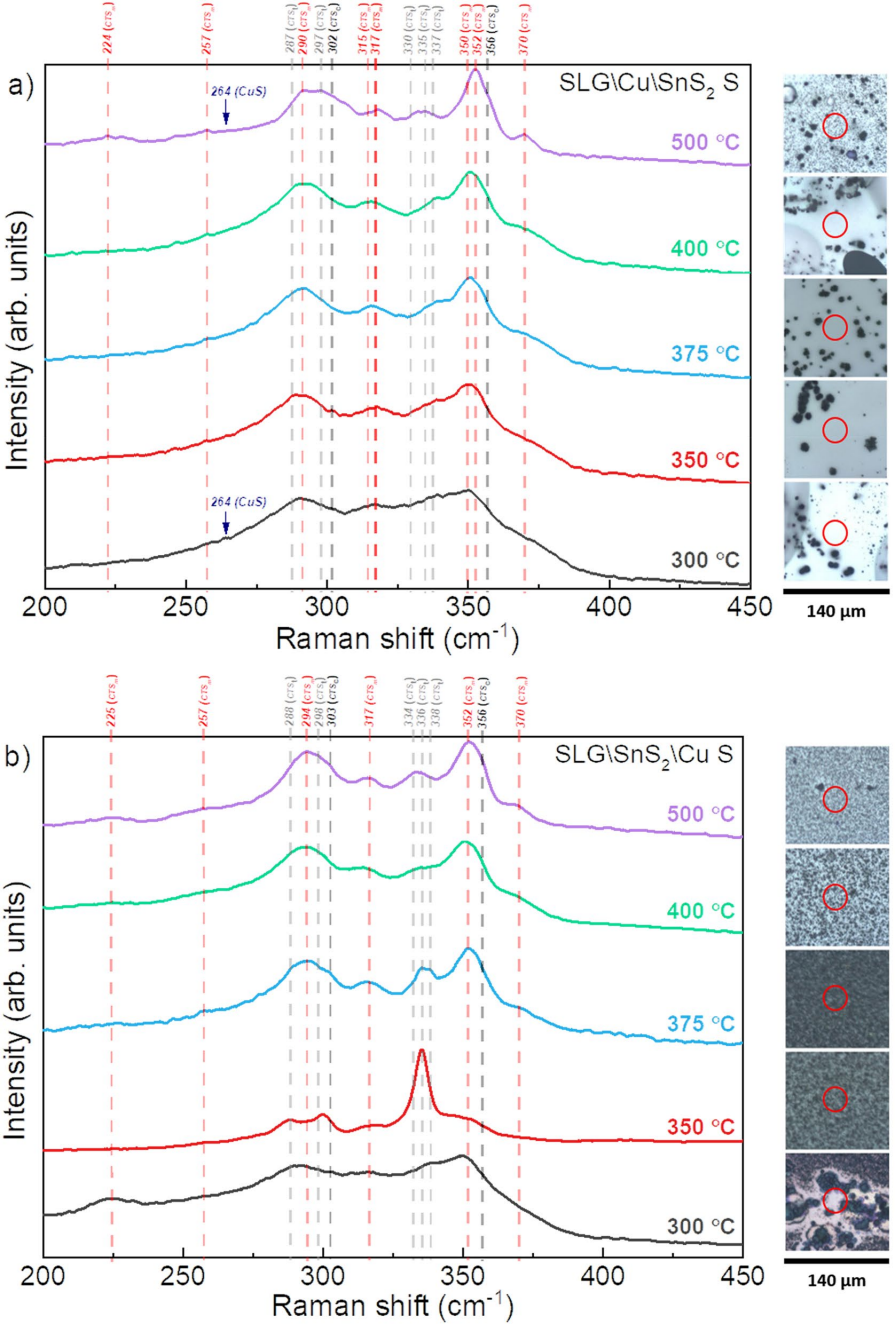
Raman spectra of the CTS films annealed under S atmosphere at different temperatures, measured using a 633 nm wavelength. (**a**) SLG\Cu\SnS_2_ and (**b**) SLG\SnS_2_\Cu stacks. The dashed vertical lines represent the Raman shifts of all identified or possible phases. On the right side, the relative optical images with the measured spot for each sample are shown.

**Figure 6:**
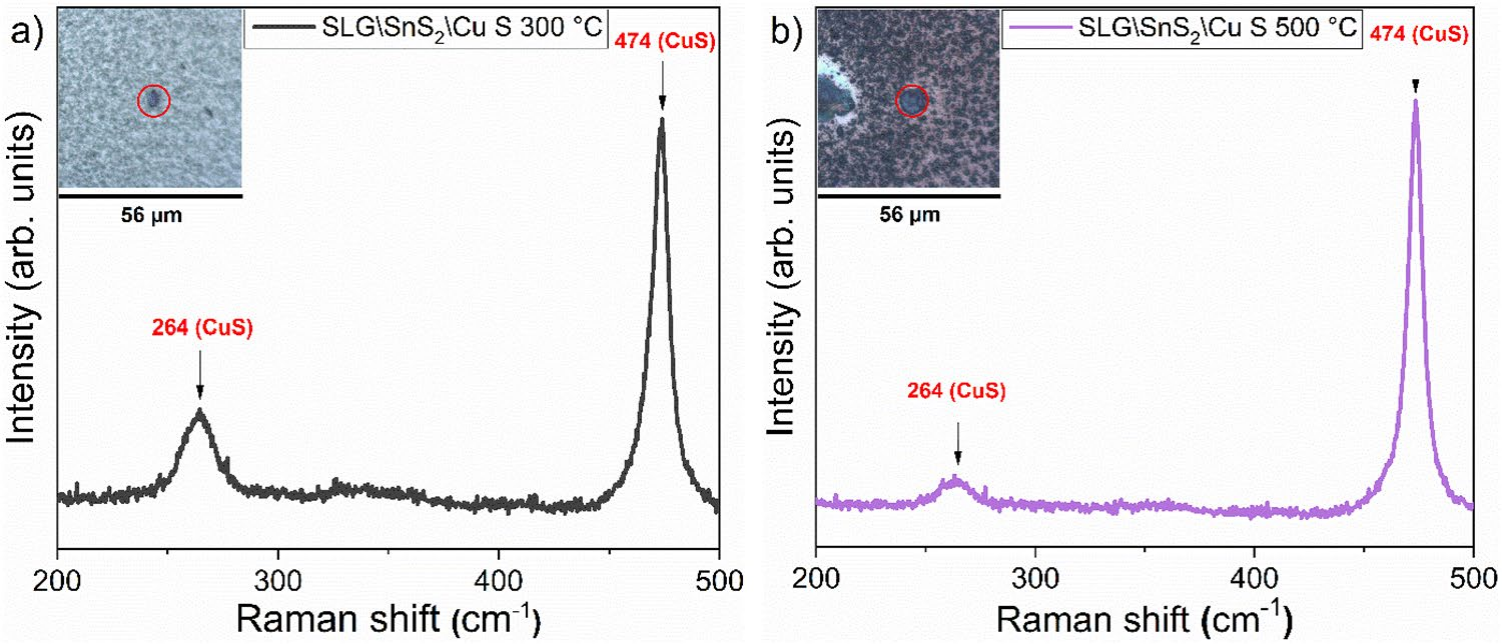
Raman spectra of the CTS samples measured using a 325 nm excitation wavelength on large grains to identify the CuS secondary phase. (**a**) SLG\SnS_2_\Cu 300 °C and (**b**) SLG\SnS_2_\Cu 500 °C in S atmosphere. The inset represents the microscopic image of the measured spot.

The Raman spectra recorded on the SLG\Cu\SnS_2_ S and SLG\SnS_2_\Cu S stacks at different temperatures, are shown in Fig. 5a,b, along with the Raman microscope images of the measured spots for each sample. The stacks prepared with Cu at the bottom have a similar mixed structure regardless of the annealing temperature (Fig. 5a), with several peaks characteristic to the monoclinic (257, 290, 317, 352 and 370 cm^−1^)^58^ and the tetragonal (287, 297 and 338 cm^−1^)^17,54^ structures. Figure 5b shows the Raman spectra of the SLG\SnS_2_\Cu stack sulfurized at temperatures between 300 and 500 °C. Raman peaks of the CTS_m_ were detected at 225, 257, 294, 352 and 369 cm^−1^. The tetragonal structure, on the other hand, was identified by the peaks at 287 and 336 cm^−1^. All samples contain both monoclinic and tetragonal structures, however, as it can be seen in Fig. 5b, the monoclinic structure is the main crystal system, except for the SLG\SnS_2_\Cu S 350 °C film. This sample is dominated by the Cu_2_SnS_3_ tetragonal structure since three CTS_t_ peaks (287, 297 and 336 cm^−1^) are observed along with a CTS_m_ peak at 317 cm^−1^. It is worth mentioning that small intensities at 303 and 356 cm^−1^ belonging to the CTS_c_ were observed in almost all the samples, which might suggest the presence of this structure in very small amounts. One should note that a significant difference is observed in the Raman microscope images of the two stacks (Fig. 5a,b). The SLG\SnS_2_\Cu samples appear to be denser and more compact compared to the SLG\Cu\SnS_2_ samples, which can be explained by the evaporation of an important quantity of SnS from the samples with SnS_2_ on top. The CuS phase was detected in the two stacks in all the samples by two peaks (264 and 474 cm^−1^)^67^ when Raman spectroscopy was performed on large crystallites found on the surface of the samples annealed at low (300 °C) and high temperatures (500 °C) as shown in Fig. 6 using a 325 nm wavelength. The CuS is known to segregate on the surface in the form of easily visible large grains^68^. This confirms the conclusions of GIXRD analysis.

**Figure 7:**
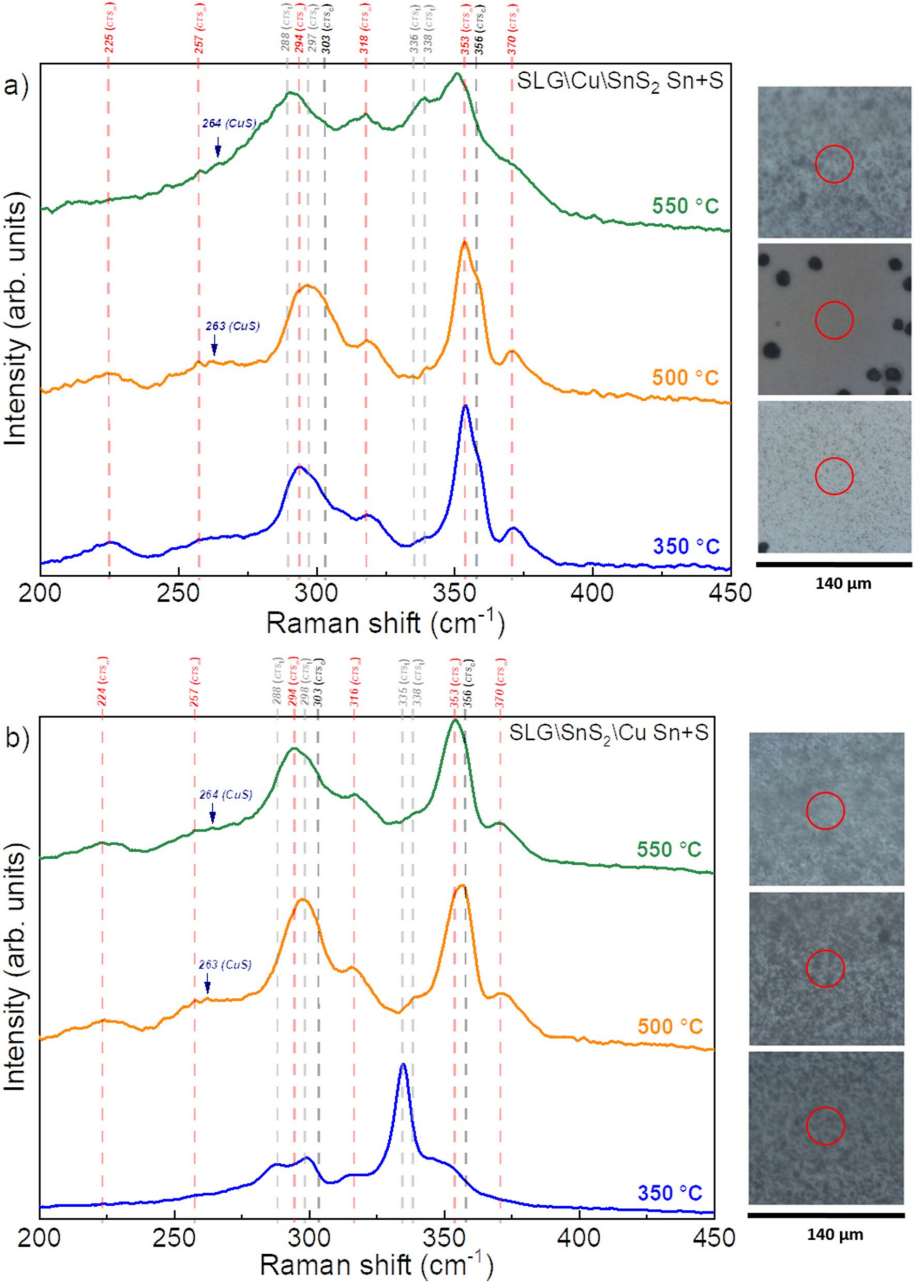
Raman spectra of the CTS films annealed under Sn + S atmosphere at different temperatures measured using a 633 nm wavelength. (**a**) SLG\Cu\SnS_2_, (**b**) SLG\SnS_2_\Cu stacks. The dashed vertical lines represent the Raman shifts of all identified or possible phases. On the right side, the relative optical images with the measured spot for each sample are shown.

The Raman spectra of SLG\Cu\SnS_2_ Sn + S and SLG\SnS_2_\Cu Sn + S samples annealed at 300, 500 and 550 °C under Sn + S atmosphere are presented in Fig. 7a,b, along with the optical images of the measured spots. It can be seen in Fig. 7a that the SLG\Cu\SnS_2_ Sn + S stack annealed at 350 and 500 °C is composed mostly of the monoclinic Cu_2_SnS_3_ phase, with the Raman peaks at 225, 257, 294, 317, 353 and 370 cm^−1^. On the other hand, the SLG\Cu\SnS_2_ Sn + S sample annealed at 550 °C contains the peaks of the CTS_m_ phase along with an intense peak at 336 cm^−1^ and a smaller peak at 288 cm^−1^ which belong to the CTS_t_ phase. The presence of the CTS_t_ peaks can be related to the evaporation of SnS at high temperature leading to an increase in copper content. Similar conclusions were drawn in a study of the effect of Cu concentration on the structure of CTS thin films^55^, where the sample with the highest concentration of copper has several peaks belonging to the monoclinic CTS structure and a strong CTS_t_ peak at 335 cm^−1^. On the contrary, the stack with Cu on top show different results (Fig. 7b). The sample annealed at 350 °C consists of a dominant CTS_t_ phase with peaks at 287, 298 and 335 cm^−1^ and a weak signal at 317 cm^−1^ of the CTS_m_ phase. The two other samples annealed at higher temperature, 500 and 550 °C, are mostly composed of the CTS_m_ identified by the peaks at 224, 257, 294, 316, 353 and 370 cm^−1^. CTS_c_ peaks at 303 and 356 cm^−1^ might exist in small quantities in all samples. One should note that the Raman spectra of all the samples annealed in Sn + S atmosphere were recorded in numerous spots, and no peak of the CuS phase was observed in any of the stacks except of a small shoulder at 264 cm^−1^ in the samples annealed at 500 and 550 °C. This can be explained by the presence of a small amount of this phase that couldn’t be detected on the sample surface in the measured spots. All the identified or possible Raman peaks in both stacks annealed at different temperatures under S and Sn + S atmospheres are summarized in Table 2.

**Table 2:** Raman peaks of the CTS_c_, CTS_m_ and CTS_t_ structures identified or possible in this work with the principal peaks being bolded.

Sample reference	CTS_c_ (cm^−1^)	CTS_m_ (cm^−1^)	CTS_t_ (cm^−1^)
SLG\Cu\SnS_2_ S 300 °C	303	290, 317, **350**, 372	337
SLG\Cu\SnS_2_ S 350 °C	303, 356	257, 290, 317, **350**, 370	287, 337
SLG\Cu\SnS_2_ S 375 °C	303, 356	257, 290, 315, **350**, 370	287, 337
SLG\Cu\SnS_2_ S 400 °C	303, 356	257, 290, 315, **350**, 370	287, 337
SLG\Cu\SnS_2_ S 500 °C	303, 356	222, 257, 290, 317, **352**, 370	297, 335
SLG\SnS_2_\Cu S 300 °C	303, 356	224, 294, 318, **352**, 371	288, 338
SLG\SnS_2_\Cu S 350 °C	–	318, 352	288, 298, **336**
SLG\SnS_2_\Cu S 375 °C	303	257, 294, 317, **352**, 371	288, 334, 338
SLG\SnS_2_\Cu S 400 °C	–	224, 257, 294, 316, **352**, 370	288, 298, 334
SLG\SnS_2_\Cu S 500 °C	–	224, 257, 294, 316, **352**, 370	**288**, 298, 334
SLG\Cu\SnS_2_ Sn + S 350 °C	303, 356	225, 257, 294, 318, **353**, 370	338
SLG\Cu\SnS_2_ Sn + S 500 °C	303, 356	225, 257, 292, 318, **353**, 370	**297**, 338
SLG\Cu\SnS_2_ Sn + S 550 °C	303, 356	225, 257, 292, 316, **353**, 370	**287**, 297, 336
SLG\SnS_2_\Cu Sn + S 350 °C	–	317	288, 298, **335**
SLG\SnS_2_\Cu Sn + S 500 °C	303, 356	224, 257, 294, 316, **353**, 370	**298**, 338
SLG\SnS_2_\Cu Sn + S 550 °C	303	224, 257, 294, 316, **353**, 370	298, 338

**Figure 8:**
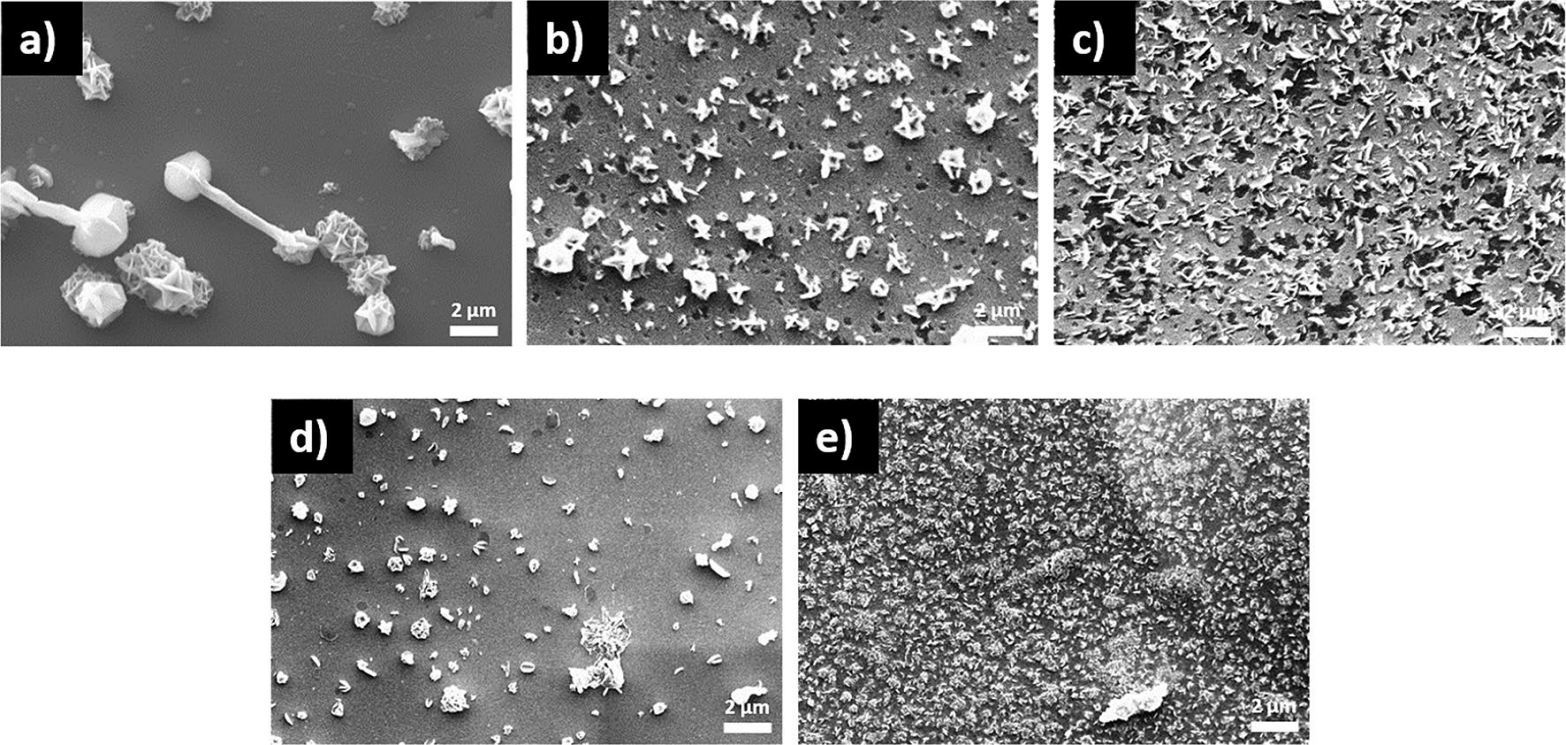
Surface SEM images of the SLG\Cu\SnS_2_ stack annealed in S atmosphere. The samples were sulfurized at (**a**) 300, (**b**) 350, (**c**) 375, (**d**) 400 and (**e**) 500 °C.

Figure 8 shows the scanning electron microscopy (SEM) images of the SLG\Cu\SnS_2_ S stack sulfurized at temperatures between 300 and 500 °C. It can be seen in Fig. 8a–e that the surface morphologies are inhomogeneous with voids (voids are visible only in Fig. 8b,c) in almost all the samples, due to the evaporation of SnS. The SEM image of the film sulfurized at 300 °C, in Fig. 8a shows a surface with grains of different shapes and sizes. As the temperature increases, surface of the films is more homogeneous and the particles become smaller and more monodispersed in size (Fig. 8b–d). The morphology of the film annealed at 500 °C, (Fig. 8e) exhibits a compact surface with small grains, however, few bigger particles are also present. This inhomogeneity in particle sizes and shapes is correlated with the existence of the CuS phase^69^ as indicated previously in the GIXRD and Raman analyses.

**Figure 9:**
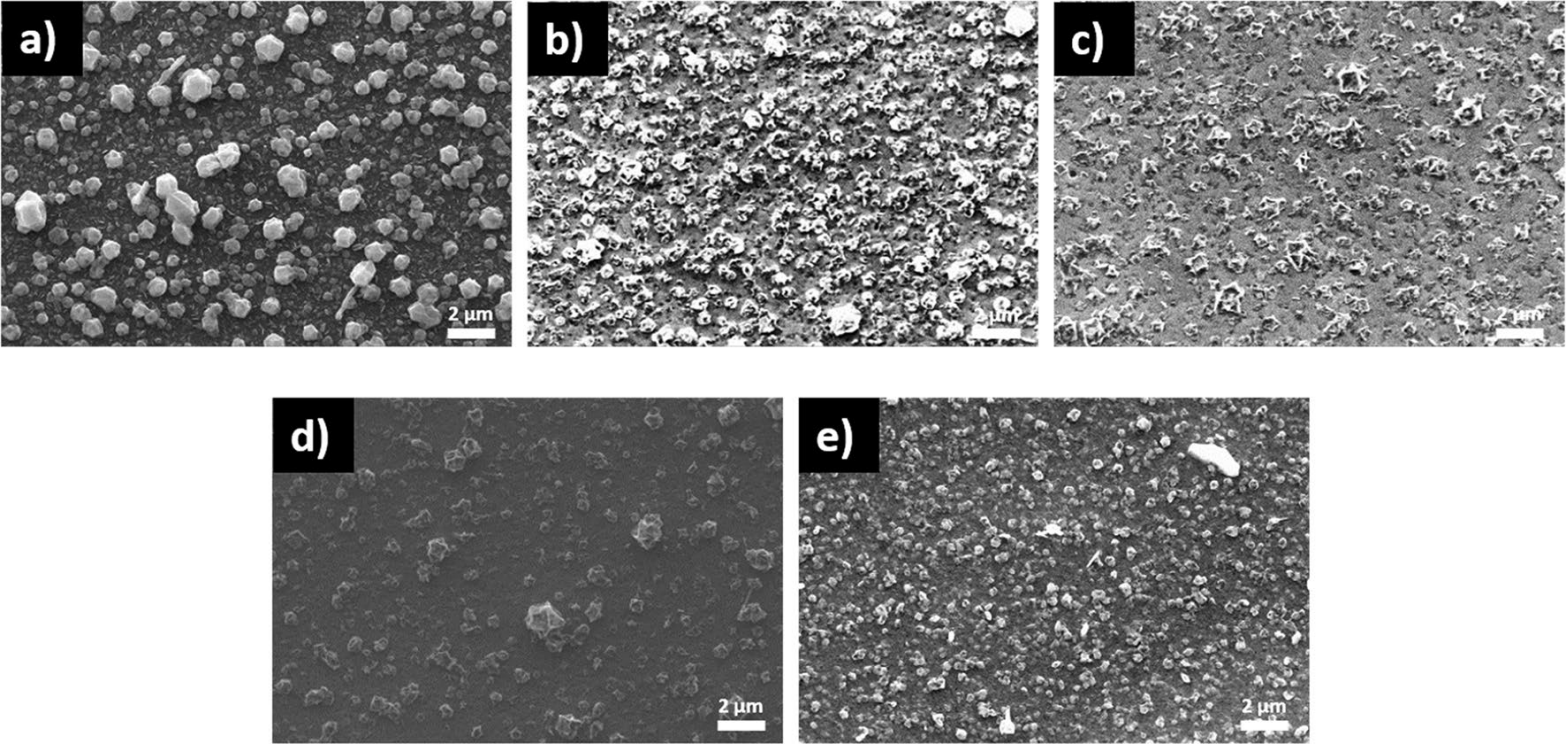
Surface SEM images of the SLG\SnS_2_\Cu stack annealed in S atmosphere. The samples were sulfurized at (**a**) 300, (**b**) 350, (**c**) 375, (**d**) 400 and (**e**) 500 °C.

The surface morphologies of the SLG\SnS_2_\Cu S films annealed at different temperatures are represented in the Fig. 9. From the SEM images it can be seen that the films are more homogeneous with fewer cracks or voids (voids are visible only in Fig. 9b,c) are present when compared to the previous stack shown in Fig. 8. As observed by GIXRD and Raman, all the samples contain large grains belonging to the CuS phase. The dominance of this secondary phase is evident in the samples SLG\SnS_2_\Cu S annealed at 300 °C. On the contrary, in the other samples, there are smaller particles rather than large particles present on the surface. The morphology of all films is compact and the size of the grains decreases by increasing the temperature. Smooth, compact and void-free morphology produces efficient charge generation and separation and it is responsible for enhanced performance of TFSC^70^.

**Figure 10:**
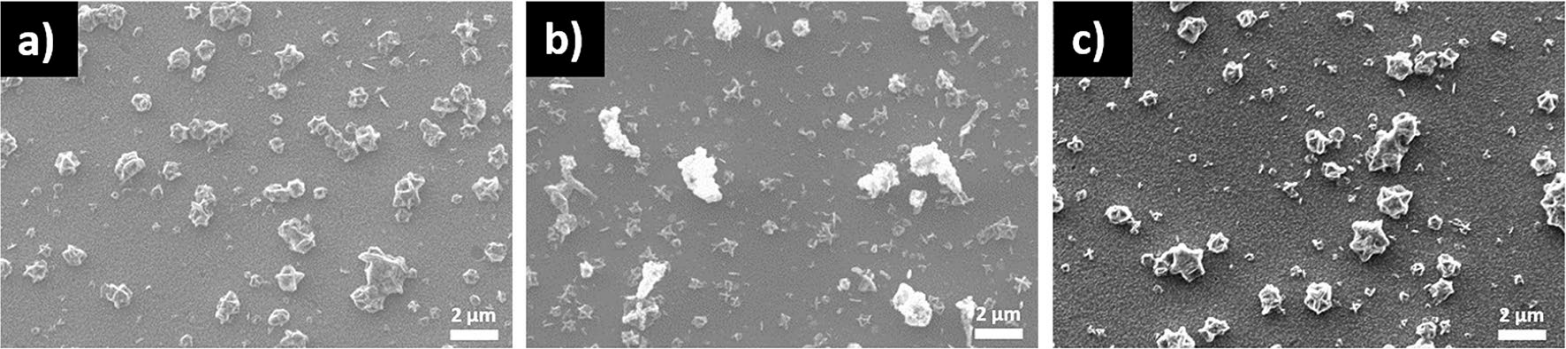
SEM images of the SLG\Cu\SnS_2_ samples annealed in Sn + S atmosphere. Annealing was performed at (**a**) 350, (**b**) 500 and (**c**) 550 °C.

Figure 10a–c illustrate the SEM results of the SLG\Cu\SnS_2_ Sn + S stack annealed at 350, 500 and 550 °C in Sn + S atmosphere. All films show sparse and dispersed surfaces, however, no voids are observed in this series of samples unlike the same stack annealed using sulfur (shown in Fig. 8). The use of an Sn + S atmosphere reduces the voids since the evaporated SnS is compensated by the Sn + S vapors^71^. The film annealed at 350 °C exhibits a homogeneous morphology with monosized grains. As the temperature increases to 500 and 550 °C (Fig. 10b,c) nonhomogeneous surfaces with different particle sizes and shapes are seen.

**Figure 11:**
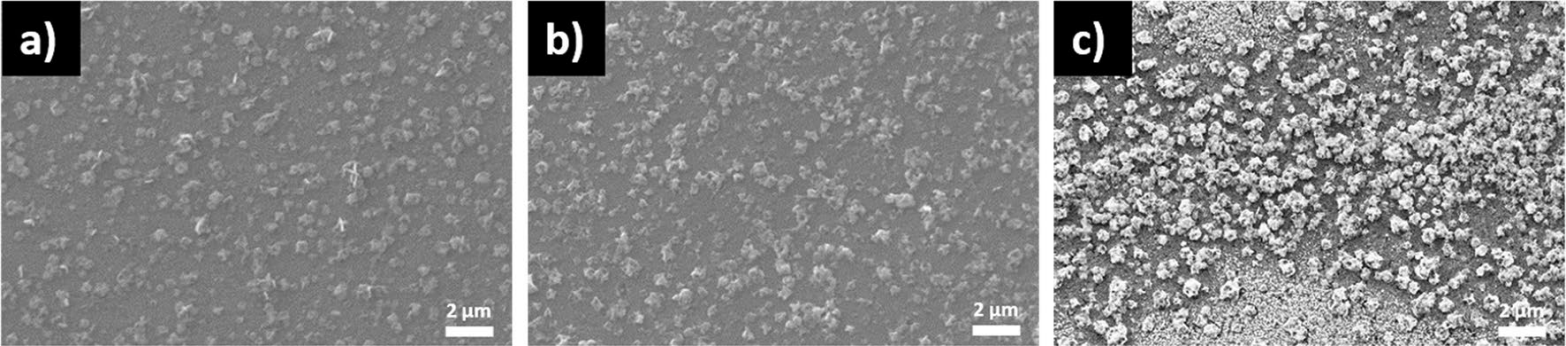
SEM images of the SLG\SnS_2_\Cu samples annealed in Sn + S atmosphere. Annealing was performed at (**a**) 350, (**b**) 500 and (**c**) 550 °C.

Figure 11a–c reveal the morphology of the CTS films from the SLG\SnS_2_\Cu Sn + S stack at different temperatures using Sn + S atmosphere in the heating treatment. All films show dense and homogeneous surfaces without voids or cracks. Also, CuS grains are not visible from the SEM images, which might be due to the small quantity of this secondary phase in the films^72^. In fact, in the GIXRD analysis the intensity of the peak corresponding to CuS in the SLG\SnS_2_\Cu Sn + S stack, is lower than in the other set of samples (SLG\Cu\SnS_2_) with the same heat treatment. Moreover, Raman spectroscopy did not identify any CuS phase in this stack.

**Figure 12:**
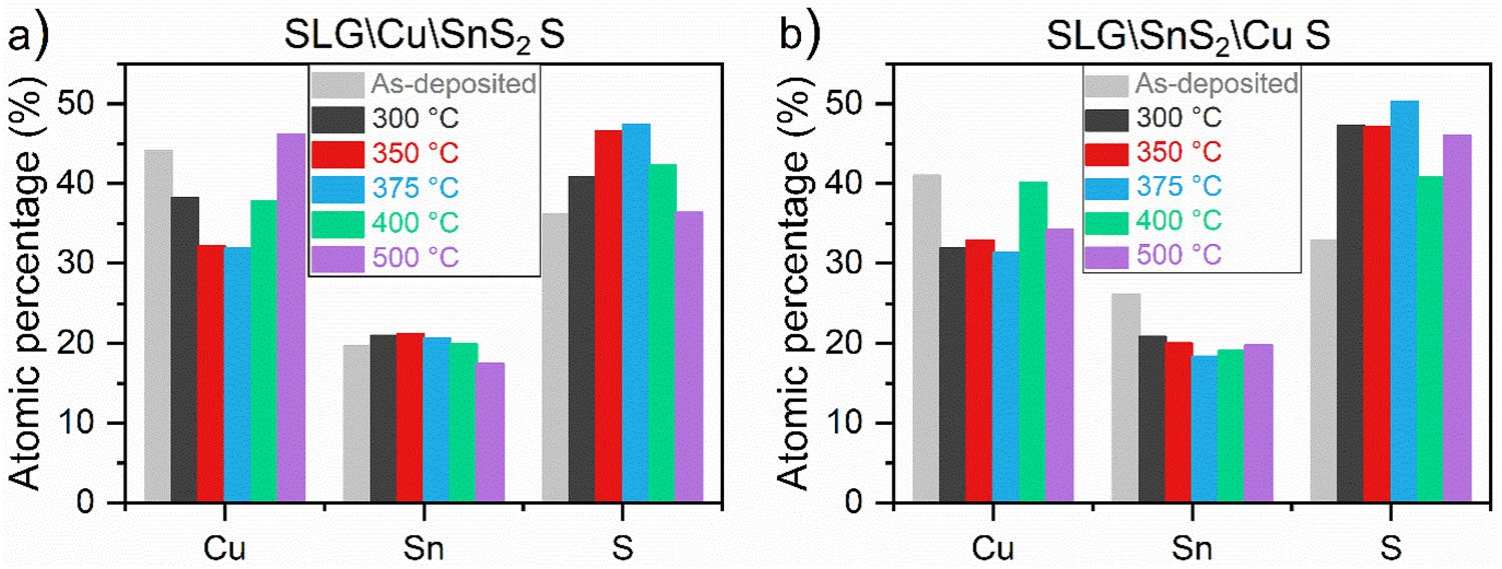
EDS results of the CTS samples annealed under S atmosphere at different temperatures. (**a**) SLG\Cu\SnS_2_ S and (**b**) SLG\SnS_2_\Cu S stacks.

Figure 12a,b present the energy dispersive X-ray spectroscopy (EDS) measurements for the as-deposited and sulfurized samples using the two stacking configurations. The results of the SLG\Cu\SnS_2_ S stack, annealed between 300 and 500 °C, are displayed in Fig. 12a. The evolution of the elemental composition of the CTS samples is characterized by a small increase followed by a decrease in Sn content when increasing the temperature. On the other hand, the copper percentage decreases at low temperatures (300, 350, 375 °C), and rises at 400 and 500 °C. On the contrary, S content is growing by increasing the temperature and reaches 50% at 400 °C (the intended percentage in stoichiometric Cu_2_SnS_3_), then slightly decreases. As observed previously, SnS is evaporated at higher temperatures in the SnS_2_-capped samples^73^, which explains the decrease in tin and sulfur content in the films annealed above 375 °C. Almost all films are Cu-rich, Sn-rich and S-poor, with the films sulfurized at 350 and 375 °C (Cu ≈ 31.32%, Sn ≈ 18.36%, and S ≈ 50.32%) being the closest to the intended stoichiometry (Cu: 33.33%, Sn: 16.67%, and S: 50%). In Fig. 12b are represented the atomic percentages of the SLG\SnS_2_\Cu S samples sulfurized between 300 and 500 °C. In this stack the elemental composition is changing arbitrarily with increasing temperature. However, almost all the samples are Cu-rich and S-poor except for the samples sulfurized at 350 and 375 °C, with the latter being closer to the CTS stoichiometry (2:1:3). The elemental composition of the SLG\SnS_2_\Cu S stack annealed at 375 °C is Cu_1.90_Sn_1.08_S_3.02_. The Cu rich phases lead to higher electrical conductivity, while the Cu poor phases affect the optical quality (bandgap) of CTS compounds. Therefore, precise elemental control is necessary to obtain a pure CTS compound^44^.

**Figure 13:**
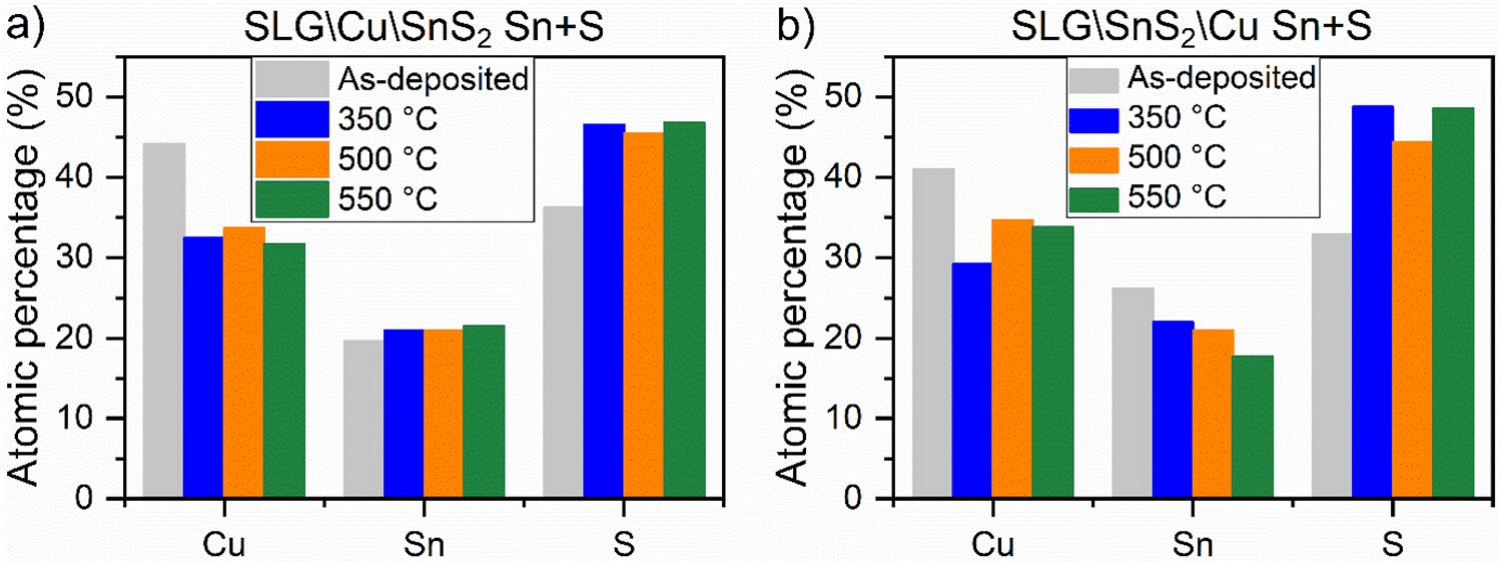
EDS results of the CTS samples annealed under Sn + S atmosphere at different temperatures. (**a**) SLG\Cu\SnS_2_ Sn + S and (**b**) SLG\SnS_2_\Cu Sn + S stacks.

Figure 13a,b show the composition of the as-deposited and annealed CTS samples in Sn + S atmosphere. The as-deposited SLG\Cu\SnS_2_ in Fig. 13a is characterized by a Cu-rich, Sn-rich and S-poor composition. By increasing the temperature between 350 and 550 °C, the copper content decreased while tin and sulfur atomic percentages increased. Copper decreased from 44.1% to stoichiometric values (between 31.6 and 33.6%). On the other hand, a tiny growth (between 20.9 and 21.5%) of the tin content is noticed, which is above the expected values. The S-poor composition observed in the as-deposited sample turns into a S close to stoichiometry in the annealed CTS films, reaching up to 47%. The sample annealed at 550 °C (Cu_1.93_Sn_1.27_S_2.80_) is the one approaching the Cu_2_SnS_3_ stoichiometry. One should note that the SLG\Cu\SnS_2_ Sn + S stack shows an enhancement in both Sn and S concentration, while annealing the same stack using only S leads to decreases of these elemental ratios. This demonstrates that using Sn + S during the heat treatment can prevent the deficiency in Sn and S^42^. The EDS results of the as-deposited and annealed SLG\SnS_2_\Cu Sn + S stack are shown in Fig. 13b. Before annealing the film is Cu-rich, Sn-rich and S-poor. At temperatures between 350 and 550 °C, the copper and tin content decreases, while sulfur is enhanced. However, it is worth mentioning that all the samples are close to the Cu_2_SnS_3_ stoichiometry, especially the SLG\SnS_2_\Cu Sn + S annealed at 550 °C (Cu_2.01_Sn_1.05_S_2.94_).

**Figure 14:**
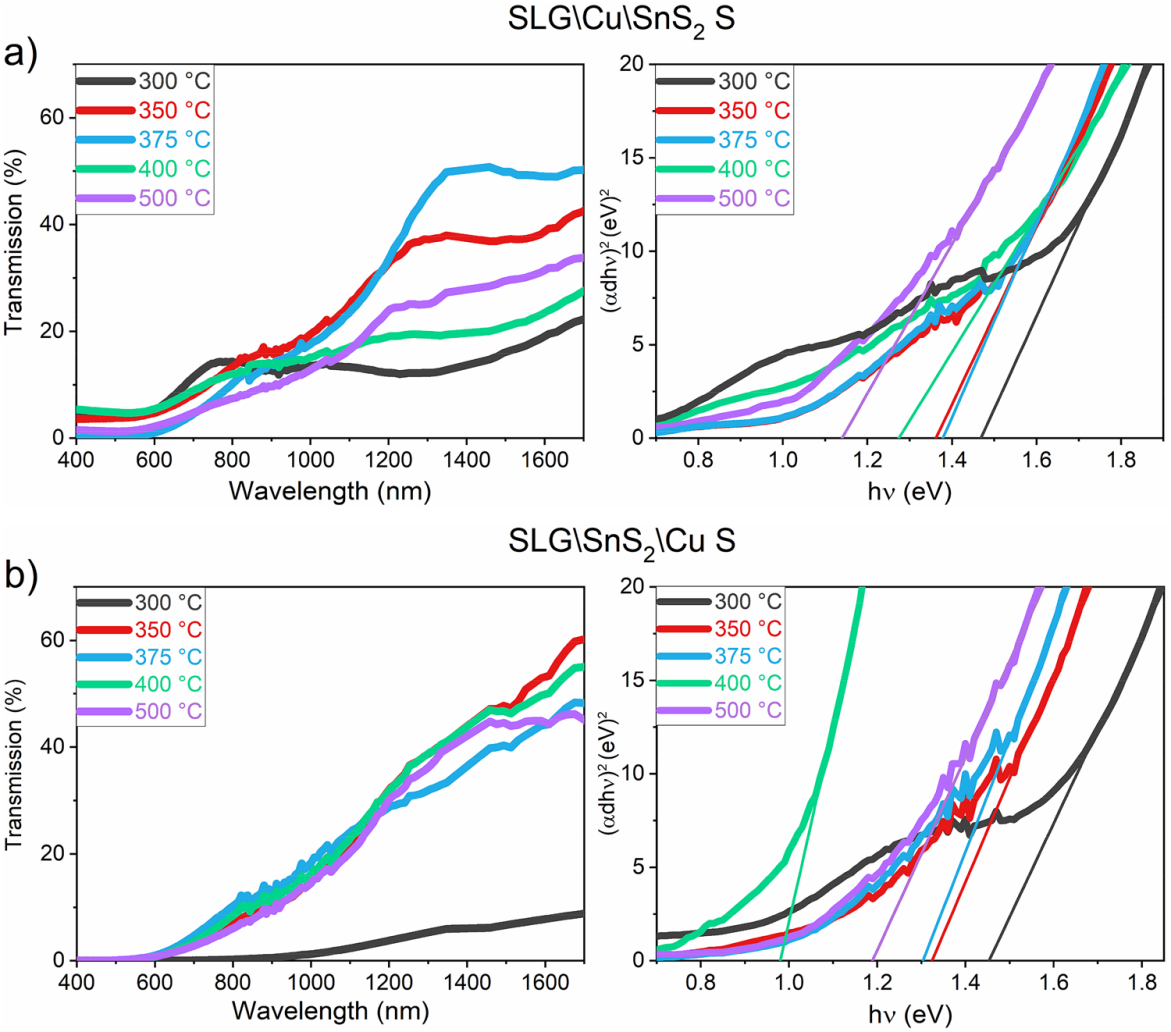
Transmission and Tauc plot of the CTS films annealed under S atmosphere at different temperatures. (**a**) SLG\Cu\SnS_2_ S and (**b**) SLG\SnS_2_\Cu S stacks.

In Fig. 14a,b are shown the band gap energies (E_g_) of the sulfurized samples, using the two stacking configurations. The SLG\Cu\SnS_2_ S samples annealed between 300 and 500 °C, show a decrease in the E_g_ values when increasing the temperature (Fig. 14a). The sample annealed at 300 °C has the highest E_g_ (1.48 eV), due to the dominance of the CuS phase, which is known to have a E_g_ between 1.8 and 2.2 eV^74^. Starting from 350 °C, the E_g_ decreases to 1.36, 1.37, 1.27 and 1.14 eV for the 350 °C, 375 °C, 400 °C, and 500 °C annealing temperatures, respectively. This happens because of the tetragonal and monoclinic mixed nature of the samples. The reported E_g_ of the monoclinic and cubic structures is around 0.95, and the tetragonal structure is characterized by a E_g_ of 1.35 eV^53,54^. As seen in Fig. 14b, the sample sulfurized at 300 °C using the SLG\SnS_2_\Cu S stack has an E_g_ of 1.45 eV. The same conclusions can be drawn here. For the CTS films sulfurized at 350 and 375 °C, the estimated E_g_ is between 1.32 and 1.30 eV, respectively. These two samples are the closest to the CTS_t_ structure as demonstrated by the structural results. The SLG\SnS_2_\Cu S sample annealed at 400 °C has the lowest E_g_ from all the samples (0.98 eV), which means that this sample is mainly composed of a monoclinic structure. At 500 °C, the E_g_ increases again to reach 1.19 eV, maybe due to few CuS particles found in this film.

**Figure 15:**
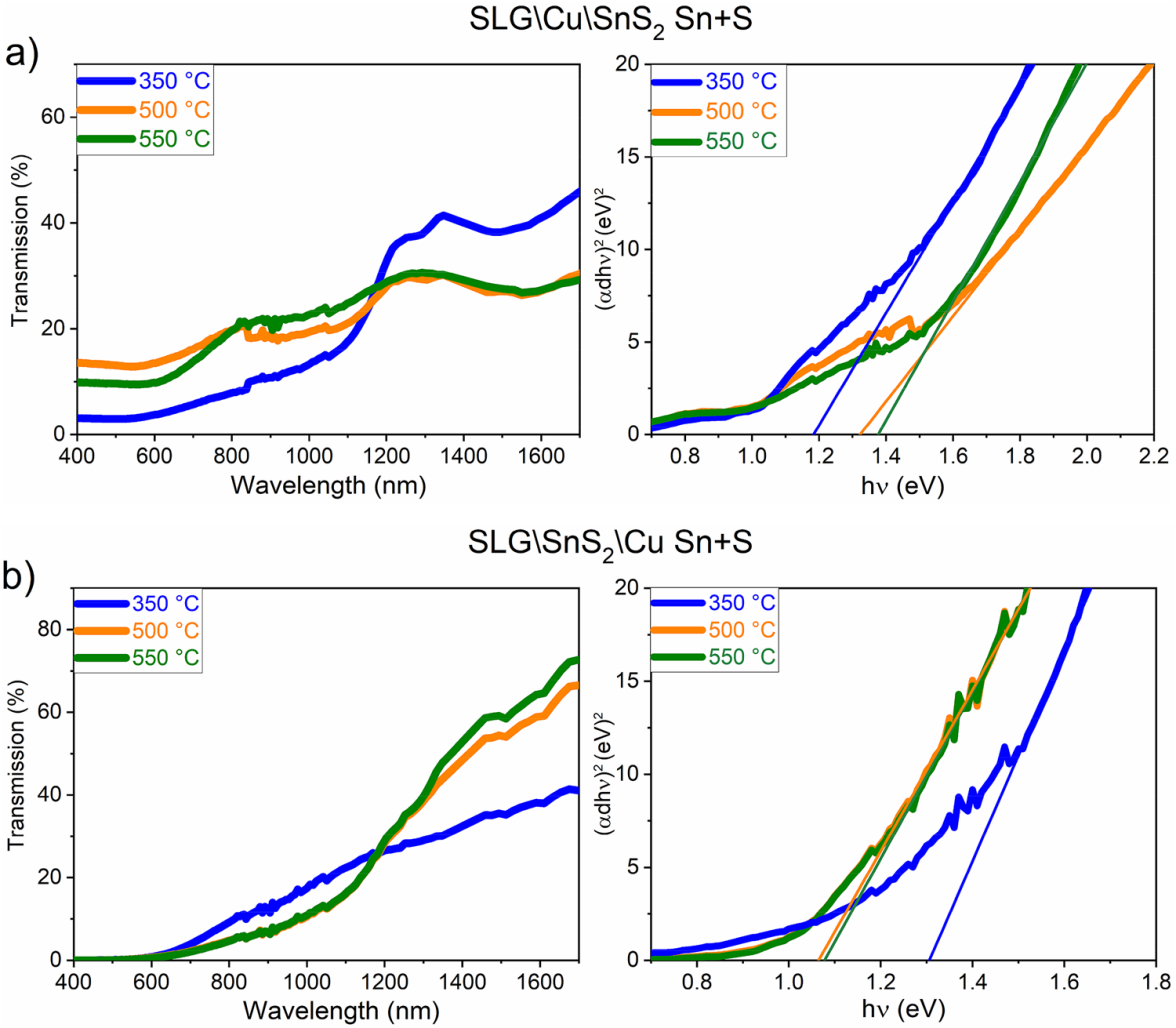
Transmission and Tauc plot of the CTS films annealed under Sn + S atmosphere at different temperatures. (**a**) SLG\Cu\SnS_2_ and (**b**) SLG\SnS_2_\Cu stacks.

In Fig. 15a,b are illustrated the E_g_ of the SLG\Cu\SnS_2_ Sn + S and SLG\SnS_2_\Cu Sn + S stacks annealed under Sn + S atmosphere. The E_g_ values, ranging from 1.18 to 1.37 eV for the samples annealed between 350 and 550 °C, are increasing with the annealing temperature in the SnS_2_-capped samples. This can be interpreted by the structural transformations of the CTS samples with the increase of temperature, since the SLG\Cu\SnS_2_ Sn + S samples are mainly composed of a mixed monoclinic-tetragonal structure. On the contrary, the SLG\SnS_2_\Cu Sn + S stack shows a decrease in the band gap values by increasing the temperature. The tetragonal structure is mostly present in the sample annealed at 350 °C, with a computed E_g_ of 1.30 eV, which is close to the reported value for this structure. The samples annealed at 500 and 550 °C are characterized by lower band gaps (1.06 and 1.07 eV, respectively), due to their polymorphic structure.

## Conclusions

In summary, eco-friendly Cu_2_SnS_3_ thin films were synthesized by annealing two stacks (SLG\Cu\SnS_2_ and SLG\SnS_2_\Cu) in two different atmospheres: S only at 300, 350, 375, 400 and 500 °C, and Sn + S at 350, 500 and 550 °C.

For the sulfurized samples, Raman spectroscopy demonstrated that all samples in the SLG\Cu\SnS_2_ S stack are polymorphic. On the other hand, the SLG\SnS_2_\Cu S stack is characterized by a tetragonal dominant structure in the film annealed at 350 °C, while the films sulfurized at higher temperature are polymorphic with more peaks belonging to CTS_m_. The GIXRD measurements revealed the formation of the h-CuS secondary phase in both stacks sulfurized at 300 °C, and starting from 350 °C, this phase is present only as a trace, regardless of the stacking order. The sulfurized SnS_2_-capped samples exhibit a poor surface morphology with multiple voids due to SnS evaporation from the top layer. This was confirmed by the gradual decrease in Sn and S content observed in the EDS analysis. On the contrary, the copper-capped films have uniform and homogeneous surfaces with nearly stoichiometric compositions. The optical measurements on the sulfurized samples showed a dependence of the band gap on the structural properties, with values between 1.14 and 1.48 eV for the SLG\Cu\SnS_2_ S stack, and between 0.98 and 1.45 eV for the SLG\SnS_2_\Cu S stack.

For the annealing under Sn + S atmosphere at different temperatures, the SLG\Cu\SnS_2_ Sn + S samples with SnS_2_ as capping layer, contain very small amount of the h-CuS phase, as inferred from GIXRD and Raman spectroscopy. SEM images show a non-uniform surface, and a small increase in the Sn and S content is observed due to the Sn + S annealing conditions. The optical energy gap is between 1.18 and 1.37 eV. On the other hand, the SLG\SnS_2_\Cu Sn + S stack annealed at different temperatures using Sn + S atmosphere, possesses overall the most adequate properties. GIXRD measurement of the film annealed at 350 °C showed a CTS phase with oriented peaks towards the tetragonal structure. This was supported by a strong peak at 335 cm^−1^ observed using Raman spectroscopy. The samples annealed at 500 and 550 °C are polymorphic with the main Raman peaks belonging to the CTS_m_ structure. The surface morphology is dense and compact in all films and the elemental compositions are the closest to stoichiometry, with E_g_ between 1.06 and 1.30 eV.

This new synthesis strategy has revealed that Cu_2_SnS_3_ films can be obtained from two different stacking configurations using Cu and SnS_2_ sputtering targets. The surface morphology can be enhanced using a Cu-capped layer to avoid SnS evaporation, the structural parameters can be controlled to obtain the desired crystalline phase by varying the temperature, while Sn + S annealing is found to be beneficial against SnS losses and helps obtain the intended stoichiometry. These findings are helpful for the synthesis of CZTS films, by the solid reaction of an additional ZnS layer on top of CTS films.

## Materials and methods

**Figure 16:**
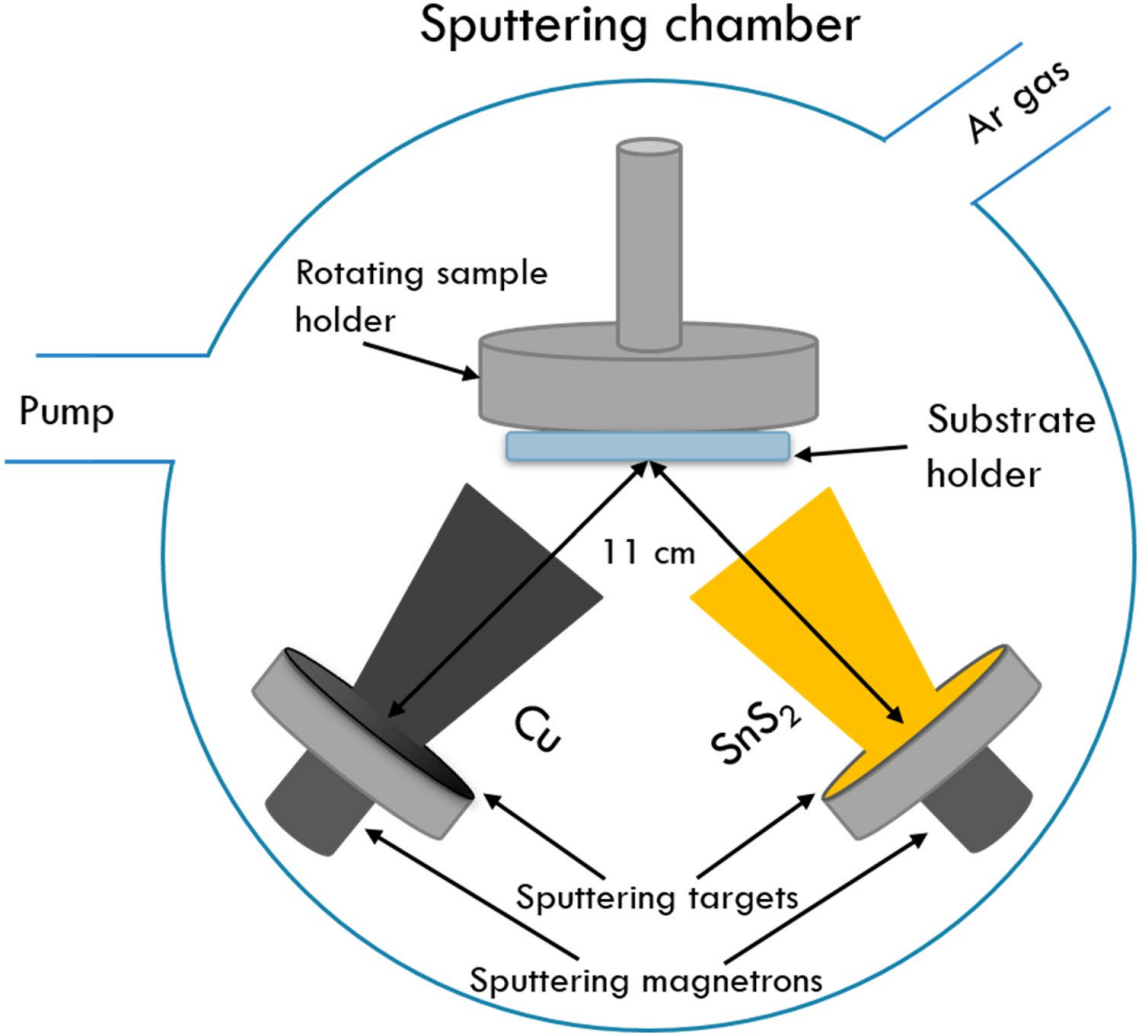
Schematic diagram of the deposition chamber.

The thin films were synthesized onto soda lime silica glass (SLG) substrates using two sets of magnetron sputtering depositions. Two stacking configurations were obtained. For the first stack, SLG\Cu\SnS_2_, the Cu layer was sputtered first, followed by the deposition of a SnS_2_ film on top. For the second stack, SLG\SnS_2_\Cu, the SnS_2_ layer was sputtered first, followed by the deposition of a Cu film on top. The films were prepared at room temperature by radio frequency (RF) magnetron sputtering (3G Circular Gencoa) using SnS_2_ and Cu sputtering targets (Mateck Gmbh, 99.99% purity). The stacked films were deposited without breaking the vacuum. The base pressure inside the deposition chamber was 4 × 10^–6^ Torr before deposition and 5 × 10^–3^ Torr during deposition, in an Argon environment with a gas flow of 30 standard cubic centimeters per minute (sccm). The substrates were continuously rotated during deposition and placed at a distance of 11 cm from the sputtering targets. Before depositions, the sputtering rates of the magnetrons were optimized using an Inficon Q-bridge deposition monitor connected to a quartz microcrystal. The sputtering power was set at 50 W for Cu, and at 25 W for SnS_2_, leading to a sputtering rate of 0.85 Å/s for Cu and 0.67 Å/s for SnS_2_, respectively, using two RF—T&C Power Conversion Inc., Model AG 0313 sources. Prior to the deposition process, a 10 min pre-sputtering was performed on each target to eliminate any undesired impurities from the target surfaces. During deposition, the Cu target was sputtered for 1176 s to obtain a 100 nm thickness Cu film and the SnS_2_ target was sputtered for 3720 s to obtain a 250 nm thickness SnS_2_ film. A schematic of the sputtering chamber and the deposition parameters are presented in Fig. 16 and Table 3.

**Table 3:** Deposition parameters.

	Target diameter (mm)	Subtrate holder rotating speed (rpm)	Distance from Sputtering target to substrate holder (cm)	Applied power to target (W)	Sputtering rate (A/s)	Sputtering time (s)	Ar gas flow (SCCM)	Deposition pressure (Torr)
Cu	50.8	12	11	50	0.85	1176	30	5 * 10^–3^
SnS_2_	50.8	12	11	25	0.67	3720	30	5 * 10^–3^

The as-deposited samples were then loaded into a quartz tube with sealing flanges inside a GSL 1600X tubular furnace, and annealed at different temperatures for 10 min with a heating rate of 10 °C/min and a cooling rate of 5 °C/min. Several results have reported that the sulfurization time plays a crucial role in controlling the elemental Sn loss from the absorber compound. Prolonged sulfurization leads to higher Sn loss^75^. The SLG\Cu\SnS_2_ and SLG\SnS_2_\Cu stacks were annealed in two different atmospheres, using as source: a sulfur powder (S) only, or a mix of tin plus sulfur powders (Sn + S). An Argon flow of 10 sccm was used to ensure the reactive gasses transport to the samples surface. Samples were annealed at temperatures between 300 and 550 °C, since above this temperature the formation of non-compact morphology attributed to the elemental Sn loss was reported^76^. The samples were named according to the stacking order, annealing atmosphere and temperature.

The structural investigations were performed by grazing incidence X-ray diffraction (GIXRD) at an incidence angle of 0.3°, using a Rigaku SmartLab diffractometer in a parallel beam configuration, provided with Cu Kα radiation (λ = 1.54178 Å) and HyPix-3000 2D Hybrid Pixel Array Detector (in 0 D mode). For Raman spectroscopy a LabRAM HR Evolution Raman spectrometer from HORIBA Jobin–Yvon, equipped with a confocal microscope and a He–Ne laser, using red and UV excitation wavelengths of 633 and 325 nm, was employed. The laser was focused using an Olympus 100× objective on the sample surfaces. The surface morphology and elemental composition of the films were measured using a Zeiss EVO 50 XVP scanning electron microscope (SEM) coupled with a Bruker Quantax 200 detector energy dispersive spectrometer (EDS). Optical transmission measurements were carried out by a V-Vase Woollam Spectroscopic Ellipsometer accessorized with a high-pressure Xenon discharge lamp integrated in a HS-190 monochromator. The transmittance was measured by conventional spectroscopy in specular mode, and the optical band gaps were calculated using the following equations^77^:

1 $$\upalpha = - { 1}/{\text{d ln }}\left( {\text{T}} \right)$$

2 $$\upalpha {\text{h}}\upnu = {\text{ A }}\surd \left( {{\text{h}}\upnu {-}{\text{E}}_{{\text{g}}} } \right)$$

where α is the absorption coefficient, hν is the photon energy, A is the constant of proportionality, d is the film thickness (which is approximately 500 nm for all films), E_g_ is the optical band-gap, and T is the recorded transmittance. The E_g_ was estimated using the Tauc plot by plotting (αdhν)^2^ vs. (hν) near the absorption edge. Since the thickness of the films is around 500 nm, a value of 20 for the product (αdhν)^2^ is corresponding to an absorption coefficient of 2.3 × 10^4^ cm^−1^. 10^4^ is a reference α value close to the band gap. Thus, the fitting of the experimental points should be performed in the range 0 to 20 in (αdhν)^2^.

## Data Availability

The data presented in this study are available on a reasonable request from the corresponding author.
